# Inactivation of Lipase and Lipoxygenase of Wheat Germ with Temperature-Controlled Short Wave Infrared Radiation and Its Effect on Storage Stability and Quality of Wheat Germ Oil

**DOI:** 10.1371/journal.pone.0167330

**Published:** 2016-12-09

**Authors:** Bo Li, Lina Zhao, Hongjian Chen, Dewei Sun, Boxin Deng, Jinwei Li, Yuanfa Liu, Fei Wang

**Affiliations:** 1 State Key Laboratory of Food Science and Technology, Synergetic Innovation Center of Food Safety and Nutrition, School of Food Science and Technology, Jiangnan University, Lihu Avenue, Wuxi, Jiangsu, P.R.C; 2 School of Food and Biological Engineering, Jiangsu University, Zhenjiang, Jiangsu, P.R.C; 3 Jiangsu Berkgen Bio-Pharmaceutical Co., Ltd, Hanjiang District, Yangzhou, Jiangsu, P.R.C; College of Agricultural Sciences, UNITED STATES

## Abstract

Wheat germ (WG) is quite susceptible to deterioration due to the presence of lipase (LA) and lipoxygenase (LOX). Therefore it is indispensable to adopt a stabilization step to decrease the activity of LA and LOX while retaining a maximum level of nutrients. But over-drying can make foodstuffs more susceptible to autoxidation. So a stabilization protocol for inactivating LA and LOX of WG with a temperature- controlled short wave infrared (SIR) radiation system was adopted to retard its rancidity and retain a maximum level of fat-soluble nutrients. Meanwhile, the critical storage water activity (Aw) of WG for inhibiting both hydrolytic and oxidative rancidity was appraised. Results indicate that WG irradiated at 90°C for 20 min acquired the optimal stabilization effect, and its residual LA and LOX activity were 18.02% and 19.21%, respectively. At this condition, the free fatty acids (FFA) content and peroxide value (PV) increment of WG oil at 40°C remained below 5% and 2.24 meq O_2_/kg for 60 days, respectively. The residual Aw of this WG sample was 0.13, and it is near the Aw corresponding to its monolayer. No significant decrease of fatty acids was observed during SIR processing, while about 96.42% of its original tocopherols still retained in WG treated at 90°C for 20 min.

## Introduction

Wheat germ (WG), main by-products of wheat milling with highest nutritional value, is removed from the endosperm during milling due to their unfavorable baking properties [1]. It is estimated that the annual world production of WG is about 25,000,000 tons [2]. The typical composition of WG includes 26–35% protein, 17% sugar, 4% minerals, 1.4–4.5% fiber and 10–15% oil with highly valuable ω-6 (44–65%) and ω-3 (4–11%) fatty acids, and it is a rich source of fat-soluble bioactive compounds such as tocopherols, policosanols and carotenoids [3–5]. Despite its excellent health benefits, the great majority of WG is just used as animal feed.

Widespread utilization of WG is limited by the rapid rancidity issues. WG is sensitive to rancidity during storage due to its large amount of unsaturated lipids as well as the presence of hydrolytic and oxidative enzymes such as LA and LOX [6, 7]. Glycerides, main ingredients of WG lipids, can be hydrolyzed to generate abundant free polyunsaturated fatty acids. Thereafter these free polyunsaturated fatty acids (preferred substrate for LOX) will undergo enzymic and non-enzymic oxidative spoilage, and the hydroperoxides yielded during oxidative reaction subsequently degrade to secondary oxidation products like aldehydes, ketones, alcohols, acids [8–10].

Considering the negative effects of rapid rancidity on the quality loss of WG oil (WGO) in short time, suitable techniques should be adopted immediately after milling to inhibit the enzymatic rancidity so as to extend its shelf life [11]. The most common method of WG stabilization is thermal processing including steaming [12], spouted bed [13] and microwave [14]. Non-uniform temperature distribution of microwave heating and low heat efficiency of convection heating limits their wide application in WG stabilization. Infrared (IR, wavelength: 0.78–1000 μm) heating has been considered as a potential technique for food processing, since the heat energy of IR can be absorbed by food materials directly. Compared with conventional heating technology, it has significant advantages including uniform heating, short heating time, low quality losses and energy consumption [15]. More recently, IR radiation has been reported to be a rapid and effective approach for retarding FFA development in rice bran [16, 17] and WG [18]. However, it is also reported that significant amount of tocopherols degraded by IR treatment, and this may be due to the high temperature generated during IR radiation.

On the other hand, water is another critical factor for food storage. Few studies are focused on the role of water during WG stabilization and storage. For lipids rancidity reaction, water can serve as both reactant and solvent [19]. Water can play both protective and pro-oxidative roles in lipids oxidation, oxidation rate of dehydrated foodstuff can accelerate at both of low and high Aw [20]. The monolayer of water is thought to be essential to cover the surface of lipids in specific foodstuff, preventing lipids from directly exposing to oxygen [21]. The monolayer and its corresponding Aw has been regarded to be correlated with the optimal food stability [22]. Exploring the critical storage Aw or Water content (WC) of WG contributes to improve its storage stability. Not only WG can suffer from extreme drying, but also the expense of WG stabilization can be cut down.

The main objectives of the present research were (1) to stabilize WG with temperature-controlled SIR radiation at 70–90°C for retaining its fat-soluble nutrients, mainly polyunsaturated fatty acids and tocopherols; (2) to explore the optimal storage Aw of WG; (3) to explore the effect of monolayer upon WG lipids hydrolysis and oxidation kinetics.

## Material and Methods

### Samples and reagents

Fresh WG was purchased from a local milling factory (Jiangsu Sanling Flour Group Co., Ltd, Taizhou, China) with the purpose of oil research in April 2015 and no specific permissions were required. Wheat germ is a by-product of wheat processing, which is sold as animal feed very cheaply by milling factory. We confirm that the field studies did not involve endangered or protected species. Oleic acid (99% pure) was purchased from Sigma–Aldrich (Shanghai, China), Linoleic acid (99% pure) was purchased from Fluka (Ronkonkoma, USA), Olive oil was purchased from Mueloliva (Priego de Cordoba, Spain). All other reagents were of analytical grade.

### WG stabilization

Fresh WG was irradiated with an experimental SIR equipment (35 cm width, 40 cm length, 27 cm height). Three 225-watt and three 450-watt IR vacuum tubes (dimension: 16×33 mm, wavelength: 1.0–2 μm, power density: 100 watt /cm) were fixed on the top of IR chamber (Senttech Infrared Technology Co., Ltd, Taizhou, China). WG stabilization was performed 11 cm below IR emitter at 70–90°C for 10–60 min, respectively. The temperature was adjusted by air circulation and IR power. For obtaining an uniform radiation, WG was spread out to form a thin layer of about 5 mm in round stainless steel trays.

### Aw and WC

Aw and WC of WG were measured before and after SIR radiation with a dew point hygrometer (LabSwift-Aw, Novasina, Switzerland) and a Halogen Moisture Analyzer (Model HB43-S, Mettler Toledo, Switzerland).

### Monolayer content determination

The adsorption isotherms of SIR-treated WG was determined according to the static method [23]. Briefly, ten saturated salt solutions (LiCl, CH_3_COOK, MgCl_2_, K_2_CO_3_, Mg(NO_3_)_2_, NaBr, CuCl_2_, NaCl, (NH4)_2_SO4 and KCl) were involved in this study with Aw ranging from 0.112 to 0.891 at 40°C. The monolayer water content of SIR-treated WG was calculated according to the GAB mathematical model [24] as below:

$$M_{e}=\frac{X_{m}CKAw}{\left(1\;\text{-}\;KAw)(1+(C\;\text{-}\;1)KAw\right)}$$

Where Me is the water content (%_db_), Aw is the water activity, Xm is the water content of the monolayer (%_db_), C is the constant related to monolayer adsorption heat, and K is the constant related to multilayer adsorption heat.

### LA activity determination

LA activity of WG was measured as our previous method [25]. Relative LA activity (%) = (residual LA activity /LA activity of raw WG)×100.

### LOX activity determination

LOX activity of WG was measured as our previous method [26]. Relative LOX activity (%) = (residual LOX activity /LOX activity of raw WG)×100.

### WGO preparation

Briefly, 30 g of ground WG samples (sieved with 30 mesh) was extracted with 300 mL n-hexane in conical flask with stopper (500 mL), then the flask was purged with nitrogen for 30 seconds and vibrated on a shaking water bath at 30°C for 2 h and filtered through a Buchner funnel quickly, the extraction procedures was repeated twice. The solvent was removed with a rotary evaporator at 40°C for 30 min. The residual WGO was nitrogen-filled packaged and stored at -40°C for further analysis.

### Accelerated storage experiments

The storage experiment was conducted from April to June of 2015, totally 60 days. Briefly, raw and SIR-treated WG samples (100 g) were sealed in tinfoil pouches and stored at 40°C for 60 days at relative humidity of 85%. The storage stability of lipids in germ samples was evaluated by the changes of FFA content, PV, conjugated diene acid (CD) content and p-Anisidine value (pAV) during storage. The content of FFA, PV, CD and pAV of WG was measured according to the AOCS official method Ca 5a-40, Cd 8–53, Ti 1a-64 and Cd 18–90, respectively at the time interval of every 15 days. The lipids hydrolysis and oxidation reaction kinetic analyses of WG were performed based on FFA and PV increment during storage, and the zero-order rate constants (K_FFA_ and K_PV_) were derived. The Aw, WC, LA and LOX activity of raw and SIR-treated samples were measured before and after storage, respectively.

### Tocopherols content determination

Tocopherols (α, β, γ and δ) content of WGO was quantified according to the method of Ng, Lean-Teik [27] with some modifications. Briefly, 1.0 g of WGO sample was dissolved in 10 mL hexane, filtered (0.45 μm) and injected (20 μL) into a HPLC system (MD-910 multi wavelength detector, PU-980 pump, DG-980-50-3 line degasser, and LG-980-02 ternary gradient unit, Jasco, Japan) with a silica gel column (Nucleosil Si, 5 μm i.d., 200 × 4mm). The mobile phase was n-hexane/ isopropanol (98.5:1.5, v/v) and the flow rate was 1.0 mL/min. Individual tocopherols quantified as mg/kg of oil with corresponding external standards.

### Fatty acid composition determination

The fatty acid composition of WGO was analyzed according to the method of Shin and Godber [28] with modifications. 50 mg of WGO was mixed with 2 mL of 0.5 mol/mL methanolic NaOH and heated at 70°C for 60 min, thereafter 2 mL of boron trifluoride/methanol (1:3, v/v) regent was added and heated at 70°C for 10 min, the fatty acid methyl esters (FAME) was extracted with 2.0 mL of n-hexane. 2μL of FAME was used for analysis. FAME was identified and quantified with a Agilent 7820 gas chromatograph (Agilent Corp., USA) equipped with a flame ionization detector (FID) and a TRACE TR-FAME capillary column (60 m × 0.25 mm × 0.25 μm, Thermo Fisher, USA). The oven temperature was set to rise from 110 to 250°C at a rate of 8°C/min and maintained at 250°C for 10 min. The injector and detector temperatures were set at 250°C. Nitrogen was used as carrier gas and the flow rate was 1 mL/ min.

### Statistical analysis

All the determinations were made in 3 replicates. Data were assessed on the statistical significance of p<0.05. Variance analysis was performed with SPSS statistical software (version 17.0, SPSS Inc., Chicago, IL, USA).

## Results and Discussion

### WC and Aw

**Fig 1:**
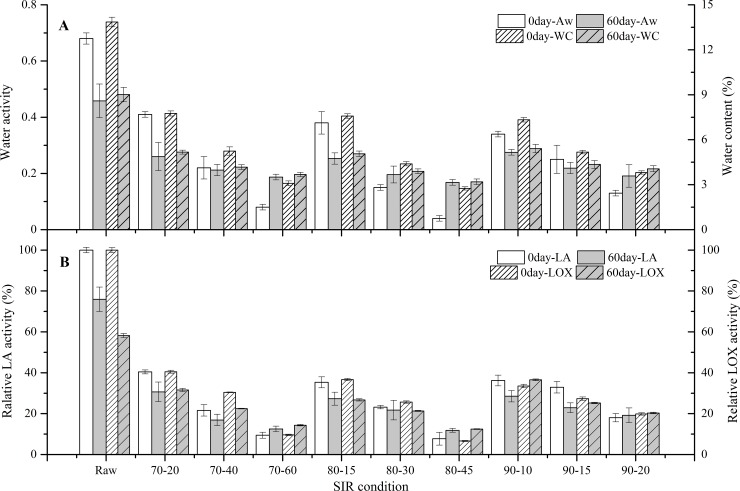
The water activity and residual enzyme activity of the WG samples before and after SIR radiation. (A)Aw and WC, (B) LA and LOX activity. (Note: x-axis values reflect SIR condition, the number before the hyphen is surface temperature: 70–90°C, the number before the hyphen is radiation time: 10–60 min).

Water plays an important role in lipids rancidity. The changes of Aw and WC of raw and SIR-treated WG during storage are shown in Fig 1(A). The initial WC and Aw of raw WG are 13.85% and 0.68, respectively. It is obvious that the Aw and WC of WG significantly decreased with the processing temperature and duration time during SIR radiation (p<0.05). The Aw of WG samples treated at 70°C for 60 min, 80°C for 45 min and 90°C for 20 min decreased to 0.08, 0.04 and 0.13, respectively. Their corresponding WC were 3.09, 2.75 and 3.81%, respectively. It is reported that Aw of WG dried at 200°C for 6 min with spouted bed decreased to 0.4. After storage at 40°C for 60 days, the Aw and WC of most germ samples with a WC above 4% significantly decreased (p<0.05), and it may be related to the water permeability of the packaging material, thus these germ samples desorbed moisture from WG to environment. While those germ samples with a WC below 4% are prone to sorb moisture from environment. There was no significant difference of the Aw among most SIR-treated germ samples after 60 days, and they were in the range of 0.17–0.28 (p<0.05).

### LA and LOX activity

Lipids of WG can be hydrolyzed by LA to generate FFA during storage, and the FFA released can further be oxidized enzymatically and non-enzymatically. LOX is responsible for catalyzing the reaction between oxygen molecule and polyunsaturated fatty acids, mainly linoleic acid [29]. IR treatment can depress LA activity of naked oats [30] and LOX activity of soybean samples [31]. The changes of LA and LOX activity are shown in Fig 1(B). During SIR radiation, LA and LOX activity of WG significantly decreased with the processing temperature and duration time (p<0.05). The residual LA activity of the WG samples treated at 70°C for 60 min, 80°C for 45 min and 90°C for 20 min decreased to 9.43, 7.72, 18.02% respectively, and the corresponding LOX activity decreased to 9.64, 6.61,19.21%. The residual LA activity of WG roasted with spouted bed at 200°C for 6 min, rotary drum at 130°C for 5 min and air-circulation oven at 150°C for 25 min were above 35%, 45.9% and 84.7%, respectively. The residual LA and LOX activity of flameless catalytic infrared treated WG for 6 min decreased to 7.94% and 14.33%. By comparison, the inactivation effect of LA and LOX in WG with SIR upon was close to or better than the previous technologies at lower temperature, and it contributes to reduce the quality loss of WG. However, the LA and LOX activity of most germ samples significantly decreased after storage (p<0.05), and this may be attributed to the water desorption phenomena. It is reported that there is a significantly positive correlation between enzyme activity and Aw in foodstuff [32]. While the LA and LOX activity of germ samples treated with SIR at 70°C for 60 min, 80°C for 45 min and 90°C for 20 min increased after storage, and it was in agreement with the results of Aw. Based on the early investigations of West and Cruz [33] and Loeb, Morris [34], the FFA content of dried rice bran began to increase again after absorbing moisture. It reveals that it is hard to irreversibly destroy LA and LOX of WG with low moisture. The LA and LOX of SIR-stabilized WG could regenerate after water adsorption and decrease after water desorption during storage. So the mechanism of WG stabilization with SIR radiation may be ascribe to Aw control.

### Storage stability

#### FFA content

**Fig 2:**
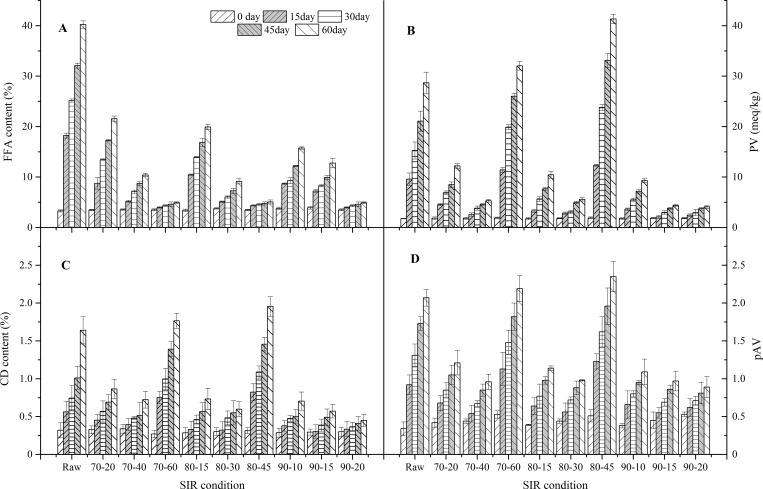
The hydrolytic and oxidative stability of WG samples during storage. FFA content, (B) PV, (C) CD and (D) pAV. (Note: x-axis values reflect SIR condition, the number before the hyphen is surface temperature: 70–90°C, the number before the hyphen is radiation time: 10–60 min).

FFA content is a typical indicator for estimating hydrolytic rancidity extent of lipids. FFA content of WG samples during storage are shown in Fig 2(A). There is no significant difference of the initial FFA content between raw and SIR-treated germ samples (p>0.05). The FFA content of raw germ samples increased rapidly from 3.29% to 40.24% oleic acid at 40°C during storage, and its FFA accumulation was considerably higher than SIR-treated WG samples (p<0.05). The FFA increment of the WG samples treated with SIR at 70°C for 60 min, 80°C for 45 min and 90°C for 20 min remained below 5% oleic acid at 40°C for 60 days. If the FFA content of 5% is chosen to be the upper limit for acceptable WGO, these SIR-treated samples can acquire a shelf life of more than 60 days at 40°C, and less than 4 days for raw germ samples at the same condition. So irradiating with SIR at 70°C for 60 min, 80°C for 45 min and 90°C for 20 min were all suitable for inhibiting hydrolytic rancidity of WG, and the minimal FFA accumulation was observed in the germ samples treated at 90°C for 20 min.

#### PV

Oxidation extent of WG was evaluated by measuring PV, CD content (primary oxidation products) and pAV (secondary oxidation products). PV of germ samples during storage are shown in Fig 2(B). The initial PV of WG was very close to each other, irrespective of different SIR processing conditions (p>0.05). However, PV of raw WG samples increased significantly from 1.77 to 28.68 meq O_2_/kg WGO after storage for 60 days at 40°C (p<0.05). The rapid oxidation of raw WG may be ascribed to the existence of abundant FFA hydrolyzed by LA, and the free linoleic acid was preferred substrate for both enzymatic oxidation and autoxidation. PV development of raw germ was considerably higher than most SIR-treated WG samples (p<0.05), except for the germ samples exposed to SIR at 70°C for 60 min and 80°C for 45 min. Over-drying can cause foodstuff more sensitive to autoxidation again. Thus, the lipids of WG can expose to oxygen directly and accelerate autoxidation of WG. If the PV of 10 meq O_2_/kg is chosen to be the upper limit for acceptable WGO, those germ samples treated at 70°C for 40 min, 80°C for 30 min, 90°C for 15 min, 90°C for 20 min all can acquire a shelf life of more than 60 days at 40°C.

#### CD content

The CD content is an excellent indicator for estimating oxidative extent of lipids rich in linoleic acid such as WGO. As shown in Fig 2(C), the CD content of raw WG increased significantly from 0.33% to 1.64% during storage (p<0.05). Linoleic acid is the excellent substrate for LOX of WG [35]. In raw WG, linoleic acid in FFA was catalyzed by active LOX to generate conjugated diene acid. Similarly, final CD content of germ samples treated at 70°C for 60 min and 80°C for 45 min were significant higher than raw WG (p<0.05), and the minimum increase of CD content was observed in the samples treated at 90°C for 20 min.

#### pAV

The pAV reflects the content of secondary oxidation products of lipids, mainly aldehyde content such as 2, 4-dienals and 2-alkenals. pAV of WG samples are shown in Fig 2(D), pAV of raw germ significantly rose from 0.34 to 2.07 during storage (p<0.05). The increasing rate of germ samples treated at 70°C for 60 min and 80°C for 45 min were significant higher than raw WG (p<0.05). This may be related to the low humidity of these SIR-treated WG samples. The SIR processing parameters of 70°C for 40 min, 80°C for 30 min, 90°C for 10–20 min are all effective for inhibiting rapid formation of aldehydes.

Combination of the results of FFA content, PV, CD and pAV, SIR radiation at 90°C for 20 min was the optimal stabilization condition for inhibiting both hydrolytic and oxidative rancidity.

### Effect of Aw upon rancidity rate

#### Monolayer content

**Fig 3:**
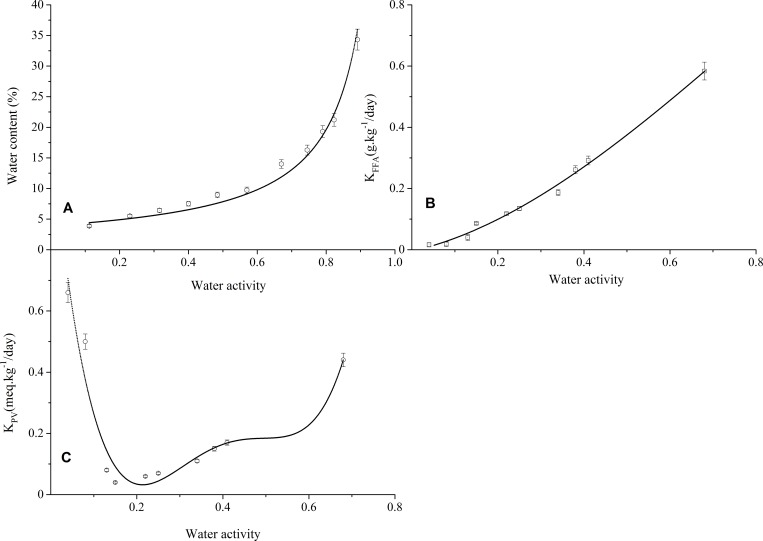
The sorption isotherms and rancidification rate of WG samples during storage. sorption isotherms, (B) hydrolytic rate and (C) oxidative rate.

The experimental adsorption isotherms data acquired at 40°C for WG samples were fitted with GAB equation, and the fitted curve is shown in Fig 3(A). The GAB equation fitted the experiment results well (R^2^ = 0.979). The Xm of WG calculated with the GAB model was 4.75%_db_ corresponding to the Aw of 0.14 at 40°C, and it is not consistent with the Xm of wheat flour at 20–65°C reported previously [36]. This may be ascribed to the different composition between WG and wheat flour. Romani, Rocculi [37] proposed the relationship between monolayer and PV of biscuits during storage. Xm is recognized to be very crucial for the physical and chemical stability of dehydrated foodstuffs, since plasticization effect usually occurs at the Aw below monolayer with leading to a lower molecular mobility and reaction rate.

#### Hydrolytic rate

The hydrolysis reaction rate constants (K_FFA_) of lipids in WG samples with different Aw during storage were calculated with the zero-order reaction rate equations, and the fitted curve is shown in Fig 3(B). The K_FFA_ is polynomially related to Aw (y = -0.46x^3^+1.12x^2^+0.31x, R^2^ = 0.989) during storage. The hydrolysis rate of lipids in WG significantly decreases with Aw declining (p<0.05). Amrani, Fayol [38] proposed that LA activity of WG was strengthened with Aw increasing. This may be due to the fact that water acts as both reactant and solvent in lipids hydrolysis reaction. Thus hydrolysis rate of lipids in WG significantly decreases due to the limited molecular mobility of LA and substrate (triglyceride). Duckworth and Smith [39] declared that solute movement was not detectable below monolayer value. Therefore, it can be suggested that decrease of Aw may be the crucial factor for retarding rapid hydrolysis rancidity of WG. Thus SIR radiation at relatively low temperature (below 90°C) is sufficient to suspending FFA accumulation in WG.

#### Oxidative rate

The oxidation reaction rate constants (K_PV_) of lipids in WG samples with different Aw during storage is shown in Fig 3(C). The K_PV_ is polynomially related to Aw (y = 76.49x^4^-121.11x^3^+67.71x^2^-15.23x+1.21, R^2^ = 0.93) during storage. The oxidative rate of WG lipids significantly decreased with the decline of residual Aw during storage till the Aw of 0.15 (p<0.05). The actual minimal K_PV_ was observed at Aw of 0.13, and it is very close to the Aw of monolayer at 40°C (Aw = 0.14). It is claimed that the optimal oxidative stability was acquired at or near the Aw of monolayer [40–42]. It seems that there is a protective effect against oxidation upon lipids at or near the monolayer, and this may be due to the limitation of oxygen molecules to unsaturated double bonds of lipids. The oxidation rate gradually increased as the Aw of WG decreased below monolayer (p<0.05), and this may be ascribed to direct contact of oxygen molecules and lipids [43]. FFA released at higher Aw is better substrate for LOX and autoxidation. Once the Aw beyond the values of the monolayer, oxidation rate of lipids in WG began increasing gradually with Aw during storage, and this is probably related to the increased mobility of residual LOX and free radicals. From the basic scientific viewpoint, the monolayer theory can be valid for determining the optimal oxidative stability of a specific product at a specific condition. It is obvious that dehydrated WG acquired the best storage stability near to its monolayer.

### WGO quality analysis

#### Tocopherols content

**Fig 4:**
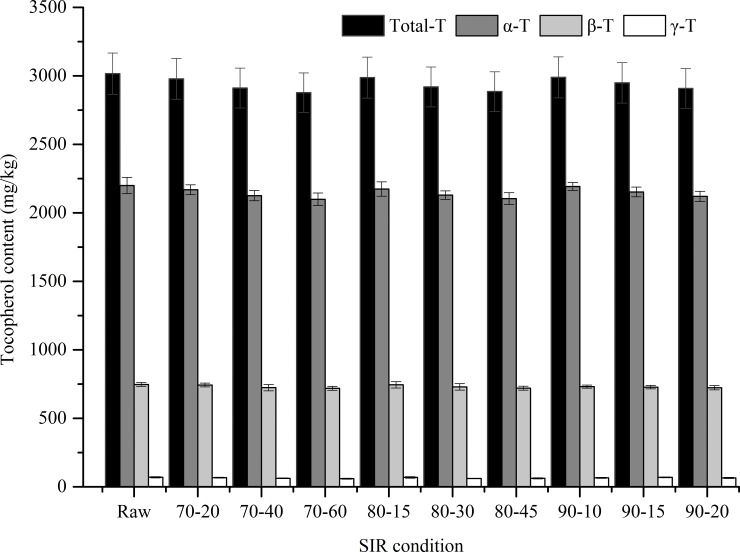
The tocopherols content of the WG samples. (Note: x-axis values reflect SIR condition, the number before the hyphen is surface temperature: 70–90°C, the number before the hyphen is radiation time: 10–60 min).

Tocopherols, a well-known lipophilic natural antioxidant, has many nutritional and healthy benefits including preventing oxidative damage and reducing blood cholesterol levels. It is reported that WGO has the highest content of α-tocopherol in vegetable oils, about 2500 ppm or even higher [44]. In this research, four tocopherol homologs (α-, β-, γ-, and δ-tocopherol) were identified and quantified. As shown in Fig 4, α-tocopherol was the predominant component (2199.20±59.68 mg/kg), followed by γ-tocopherol (747.13±13.92 mg/kg), β-tocopherol (69.79±4.51 mg/kg) and no δ-tocopherol was detected in raw WG. The individual and total tocopherols contents were insignificantly affected by the SIR radiation at 70–90°C (p>0.05). For germ samples treated with SIR at 90°C for 20 min, more than 96.43% of its original tocopherols still remained. In our previous research, it was observed that WG samples stabilized with flameless catalytic infrared (mid/far-infrared) for 6 min lost 23.82–28.75% of its original α-tocopherol. Neşe, Barış reported that rice bran treated with short wave infrared lost up to 50% of its original tocopherols in minutes, and it may be ascribed to the high surface temperature generated during radiation. Yoshida, Takagi [45] claimed that only 80% tocopherols retained in soybean samples roasted with microwave oven for 20 min. Apparently, stabilization with SIR radiation at 70–90°C contributes to retain tocopherols of WG.

#### Fatty acid composition

The main fatty acid composition of WG samples are shown in Table 1. It is reported that unsaturated fatty acids consist over 80% of the total fatty acids in WGO [46], thus WGO is used as a “premium edible oil” in Asia. In this paper, the dominant fatty acids of raw WG were linoleic (59.13±0.08%), oleic (12.89±0.09%), palmitic (16.65±0.06%), and linolenic acid (6.95±0.11%). According to Dunford and Zhang [47], the major fatty acids of WG were 55.2–56.9% linoleic acid, 14.5–14.7% oleic acid and 16.4–16.6% palmitic acid. There variation of oleic and linoleic acid content may be ascribed to different cultivars and growing districts. The effect of SIR radiation on fatty acids content of WG was found to be statistically insignificant (p>0.05). Similar findings were obtained in WG stabilization test with far-infrared, and short-infrared heating. It was found that there is no significant reduction of main fatty acid in WG heated at 130–140°C [48]. It reveals that fatty acid of WG is relatively heat stable, meanwhile SIR radiation is gentle stabilization technology for WG.

**Table 1:** Changes of fatty acid composition in WG.

Surface temperature (°C)	Processing time (min)	Fatty acid composition (%)					
		Palmitic acid	stearic acid	Oleic acid	Linoleic acid	Linolenic acid	∑PUFA
Control		16.78±0.15	0.46±0.05	12.23±0.13	60.10±0.26	7.37±0.16	66.08±0.11
	20	16.45±0.01	0.47±0.06	12.32±0.25	59.57±0.01	7.56±0.23	65.93±0.15
70	40	16.49±0.28	0.45±0.02	12.20±0.39	59.77±0.42	7.64±0.09	65.52±0.19
	60	16.58±0.21	0.45±0.05	12.26±0.23	59.67±0.54	7.21±0.07	65.65±0.12
	15	16.44±0.19	0.45±0.04	12.15±0.12	59.63±0.36	7.42±0.21	65.69±0.33
80	30	16.63±0.01	0.46±0.01	12.38±0.31	59.82±0.09	7.44±0.14	65.61±0.06
	45	16.51±0.07	0.47±0.07	12.46±0.11	59.29±0.52	7.30±0.19	65.19±0.10
	10	16.72±0.24	0.48±0.00	12.41±0.08	59.04±0.15	7.47±0.08	65.84±0.23
90	15	16.51±0.22	0.45±0.06	12.12±0.28	59.51±0.27	7.61±0.19	65.93±0.28
	20	16.34±0.10	0.46±0.04	12.25±0.37	60.15±0.58	7.67±0.20	65.52±0.27

Data are expressed as mean ± standard deviation (n = 2).

## Conclusions

In conclusion, rapid rancidity of WG could be effectively inhibited with SIR at the temperature below 90°C, and no significant decrease of fatty acids and tocopherols were observed in SIR-treated WG samples. It was found that SIR cannot irreversibly destroy the LA and LOX of WG. The possible mechanism of inhibiting the rapid rancidity of WG with SIR may be attributed to Aw control. Once the SIR-stabilized WG absorbs water from environment, its LA and LOX regenerated partly during storage. Meanwhile, the minimal oxidative rate of WG was obtained at the Aw near to the monolayer.
